# Knowledge-Based Verification of Concatenative Programming Patterns Inspired by Natural Language for Resource-Constrained Embedded Devices

**DOI:** 10.3390/s21010107

**Published:** 2020-12-26

**Authors:** Salvatore Gaglio, Giuseppe Lo Re, Gloria Martorella, Daniele Peri

**Affiliations:** 1 Department of Engineering, University of Palermo, Viale delle Scienze, Ed.6, 90128 Palermo, Italy; salvatore.gaglio@unipa.it (S.G.); giuseppe.lore@unipa.it (G.L.R.); gloria.martorella@unipa.it (G.M.); 2 Institute for High Performance Computing and Networking (ICAR), National Research Council (CNR), Via Ugo La Malfa, 153, 90146 Palermo, Italy

**Keywords:** embedded systems, wireless sensor networks, internet of things, symbolic programming, distributed programming, concatenative languages, forth

## Abstract

We propose a methodology to verify applications developed following programming patterns inspired by natural language that interact with physical environments and run on resource-constrained interconnected devices. Natural language patterns allow for the reduction of intermediate abstraction layers to map physical domain concepts into executable code avoiding the recourse to ontologies, which would need to be shared, kept up to date, and synchronized across a set of devices. Moreover, the computational paradigm we use for effective distributed execution of symbolic code on resource-constrained devices encourages the adoption of such patterns. The methodology is supported by a rule-based system that permits runtime verification of Software Under Test (SUT) on board the target devices through automated oracle and test case generation. Moreover, verification extends from syntactic and semantic checks to the evaluation of the effects of SUT execution on target hardware. Additionally, by exploiting rules tying sensors and actuators to physical quantities, the effects of code execution on the physical environment can be verified. The system is also able to build test code to highlight software issues that may arise during repeated SUT execution on the target hardware.

## 1. Introduction

**Figure 1:**
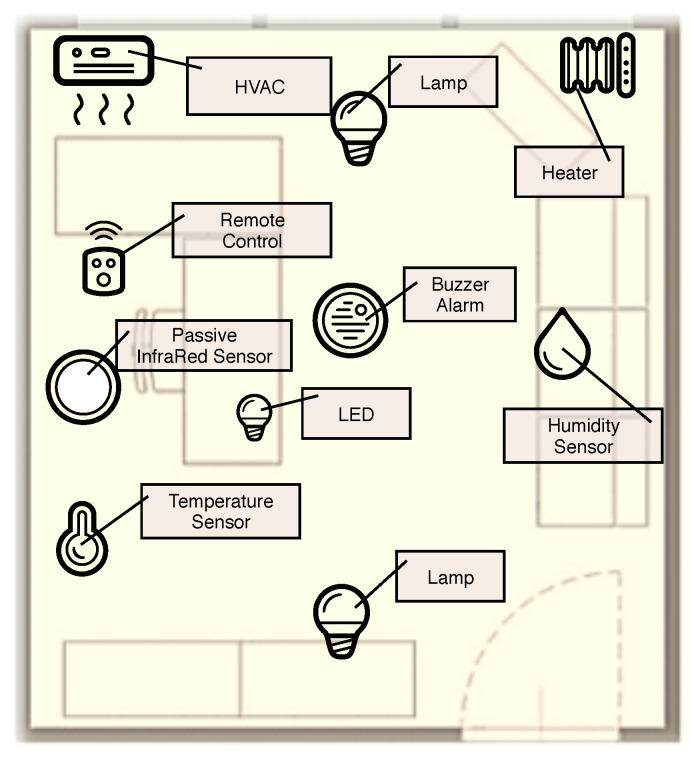
A possible reference application scenario. The physical domain is composed of objects interacting with the environment.

Developing applications interacting with physical environments, as required by the emergent Ambient Intelligence (AmI) and Internet of Things (IoT) scenarios, compels the programmer to embrace networking and computational, functional and domain aspects into a coherent whole [1]. Such an integration strongly demands novel paradigms to program heterogeneous embedded devices in a homogeneous way in order to foster interoperability [2]. In high-level AmI and IoT applications several objects, either sensors and actuators, are provided with computational abilities and interact with the environment, as exemplified in Figure 1.

These requirements collide with the scarcity of available resources on embedded devices. For instance, many approaches found in the literature adopt the Java Virtual Machine (JVM) to bring high-level symbolic programming to this class of computational units.

However, this choice often forces to sacrifice features for acceptable resource consumption, leading to partial implementations [3,4,5,6]. More generally, high-level languages are not easily applicable to embedded devices programming and resource constraints are major issues in developing and debugging such systems [7]. While simulators are widely adopted [8], designing on-board runtime verification mechanisms for resource-constrained devices demands specific efforts [9].

On the other hand, high-level abstractions may significantly support verification [10]. While a full-blown Java implementation may be out of the question for resource-constrained hardware, the simplicity of its stack-based model of computation suggests further investigations that can benefit from related work.

Assertion-based [11], model-based [12,13], and fault-injection [14] approaches have been proposed to address the problem of test oracle generation for the JVM. Even if not specifically for either embedded or runtime applications, grammar-based methodologies enriched by denotational semantics have been proposed to verify the correctness of Java high-level symbolic expressions [15]. Other works focus on test case generation techniques specifically intended for embedded systems [16,17,18,19]. Besides correct-by-construction methodologies, which generate executable code from formal specifications [20], direct generation of formal models from executable code has also been investigated [21]. In the latter case, formal specifications are defined by patterns and transformation rules are applied to the source code to produce different formal models for verification purposes.

In this paper, we propose a methodology to program resource-constrained hardware using programming patterns inspired by natural language that includes runtime verification through automated oracle and test case generation. Our approach significantly differs from all the aforementioned ones, which are intended for offline formal software verification. Moreover, the generated oracles are not exclusively bound to syntactic and semantic checks of code adhering to specified programming patterns. In fact, oracles verify that code execution leads to the expected state change of the hardware. Additionally, by exploiting rules tying sensors and actuators to physical quantities, code verification is extended to the effects of code execution on the physical environment.

The rationale behind the choice to focus on natural language patterns lays not only in the reduction of intermediate abstraction layers to map physical domain concepts into executable code but also in programming practice. In fact, the adopted symbolic concatenative computational paradigm encourages the programmer to build programs as sequences of natural language words. Moreover, while the present work does not discuss these aspects, the proposed approach converges toward the natural command control embodied by voice-based virtual assistants that more and more prominently provide the human–machine interface of AmI and IoT applications.

Our methodology supports the design of domain specific languages (DSL) with clearly defined boundaries and strong ties to natural semantics. This also avoids the implementation of complex and rigid ontologies that would need to be shared, kept up to date, and synchronized across the system.

In the proposed approach, a rule-based system integrates knowledge about physical domain concepts and properties, hardware components and programming patterns, and provides an automated oracle for a given Software Under Test (SUT). The SUT and the respective oracle are then executed on the target platform using a symbolic computational paradigm. The system is also able to generate test cases and perform repeated test execution.

The remainder of the paper is organized as follows. Related work is presented in Section 2. Section 3 focuses on the key features of the symbolic computational paradigm, while the description of the proposed rule-based system is given in Section 4.

Section 5 discusses the strategies to avoid defining patterns with ambiguous semantics in high-level applications.

Section 6 collects the experimental results. Finally, Section 7 concludes the paper.

## 2. Related Work

Current researches on IoT focus on a wide variety of novel computing applications including smart homes and automation [22,23], smart cities [24,25], personal assistants [26], and structural health of buildings [27]. To overcome the difficulties arising in programming heterogeneous devices, interpreter-based platforms have been proposed in the literature. However, most of these approaches struggle with the resource constraints of small IoT nodes. Maté [28] supports the development of applications in the form of assembly-like scripts. More recent approaches are T-RES [29] and PyFUNS [30], which are built above PyMite, a reduced Python virtual machine. Darjeeling [3] and TakaTuka [6] are virtual machines that run a small subset of the Java language. All these platforms are built above full-blown operating systems that require considerable amounts of resources. Thus, not enough space is left for applications that go beyond simple environmental monitoring [31]. The interpretation of code based on programming patterns abstracting automation rules is not totally new. For instance, the IFTTT paradigm (https://ifttt.com/discover), which enables programming heterogeneous devices through rigid reactive rules adhering to a trigger-action pattern, was adopted in several works [32,33]. Although this simple pattern covers a wide range of IoT applications, the applicability is restricted to supported devices, services, and partners, while more structured rules are difficult to implement. Similarly to this approach, we consider sensing and actuation patterns as building blocks for IoT and AmI applications, and propose a methodology to control resource-constrained hardware using imperative sentences composed of natural language words.

Abstractions naturally lead to the inclusion of verification mechanisms in the development stages as indicated by several examples the literature. ThingML [34] targets resource-constrained microcontrollers and includes concepts to describe software components using architecture models, state machines and an imperative action language. UML-based frameworks for embedded system verification that complement verification with a simulation tool have also been proposed [35,36]. In these cases, target code generation is not immediate, but it involves model-to-model transformations while verification is performed offline.

The idea to combine a rule-base logic system for verification with a language to program in a simple and intuitive way has been also proposed [37]. A simple target DSL is defined, while specifications about software, setup, and hardware are described in the knowledge base. Target code generation and automated verification relying on a logic rule base system that holds specifications about the hardware setup have also been investigated [38]. The conformance to the embedded system specification is formally verified along with the hardware configuration. Rule base systems to model new DSLs using sets of if–then rules can be also found in the literature [39]. Novel logic languages intended for formal specification and verification have been introduced to abstract, express and check desirable properties, e.g., trust, in distributed systems [40], or to specify typical embedded and Cyber-Physical System functionalities like sensing, actuation, and communication [41]. In the latter case, statistical, instead of exhaustive, model checking techniques are adopted to avoid state space explosion [42].

Besides offline verification, software instrumentation has been suggested to address online monitoring [43]. In this case, observers defined in a data-flow language are compiled into programs. Unlike our system, which binds hardware and software verification, this approach only targets software. Observers may run on a separate computer from that of the target application to check logic formulae [44]. Another approach consists in running a bytecode-based virtual machine providing a safe execution environment above an operating system on a resource-constrained device [45,46].

Other research efforts only address automatic test case generation by using different formalisms such as Statechart-like behavioral models [47], interface-automata [48], and graphical specification [49].

Differently from all these works, our approach combines a rule-base system for the automated oracle and test case generation together with on-board verification. The key idea is to bind high-level domain concepts to the related hardware operation as naturally as possible. A high-level concept exists if corresponding executable code, which produces low-level hardware effects, exists. Given an SUT expressed as a word sequence, formal specifications are used to generate the code for both on-board sentence verification, i.e., the oracle, and input test cases. Trigger-action patterns, which, as previously reported, abound in related work, are naturally included by the proposed approach. Moreover, although the oracle generation process is performed offline, it runs on the target hardware platform. Finally, also the effects on the environment of hardware-driving code are taken into account. The approach is feasible for applications including resource-constrained nodes as the underlying software platform DC4CD, which we introduced in a previous work [31], runs efficiently on this class of devices. To provide an idea of how tiny the resources offered by the target hardware are, the IRIS Mote platform, a reference platform for Wireless Sensor Networks (WSN), is based on an Atmega1281 8-bit Harvard RISC microcontroller unit (MCU) clocked at about 8 MHz with 128 KB of Flash RAM for program storage, 8 KB of RAM, and an 4 KB EEPROM. These computing resources are all that is available to run code managing a 10 bit ADC, I2C, SPI, UART and GPIO interfaces, and a IEEE 802.15.4 compliant AT86RF230 radio chip.

The idea of crafting natural language-inspired programming patterns for such machines would not even be feasible without using an efficient computational paradigm. The one discussed in the next section, on which DC4CD is based, has been proven to be effective by comparisons not only with other symbolic programming approaches for IoT and WSN development adopting interpreters like those cited above but also with environments based on cross-compilation like Contiki and TinyOS [31,50] in terms of the minimum required resources. Additionally, differently from these environments, it includes the executable code exchange as a powerful mechanism supporting the development of distributed applications despite its tiny footprint.

## 3. Computational Paradigm

The proposed approach exploits a simple stack-based symbolic computational model that can be effectively implemented even on resource-constrained devices [51].

In this model, a program *P* is a sequence of symbols $a_{1}$ $a_{2}$ …$a_{n}$. Symbols can be either numeric constants or words. Program execution is carried on evaluating the symbols in the order they are provided. While constants are simply pushed on the stack, words are used as keys to search in a structure called the word dictionary the related executable code.

For instance, execution of the code:

2 3 +

pushes 2 and 3 on the stack, then executes the word + that pops the two operands leaving the result on the stack. This simple execution mechanism, based on postfix evaluation, has been notably exploited in the Forth programming methodology [52]. Forth environments are particularly suited for embedded applications as they provide the features of a high-level interpreter and compiler, and also of an operating system, with extremely reduced resource requirements. For instance, the Forth environment we adopted for our experimental work (see Section 6) occupies about 8 KB out of the 128-KB storage memory of the IRIS mote target hardware. The effectiveness of this computational model in enabling high-level programming on resource-constrained devices has been demonstrated in the development of a software platform for distributed symbolic processing to support AmI applications on WSNs [31,53], to turn on-board hardware specifications into automatic verification code for subsystems [50] of resource-constrained devices, and to implement symbolic distributed protocols that can be automatically verified [54,55] on resource-constrained WSN nodes.

**Figure 2:**
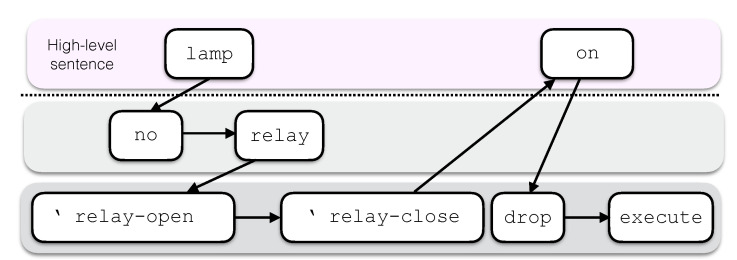
High-level words define a sentence, which can be executed and verified on the target machine. Words are defined in terms of lower-level words. Execution proceeds along the chain of definitions.

In the following, the Forth terminology is used to describe the computational paradigm. The stack discussed previously, also called parameter stack or data stack, is used for input and output parameter passing among words while a second stack, the return stack, stores return addresses, iteration control parameters and other temporary values. The word set composing the word dictionary includes:

- Built-in words that directly map to machine code. These words can be considered as terminal symbols and define operations on stack and memory as well as selection, iteration and compilation constructs;
- User-defined words that are defined as chains of words already in the dictionary. The evaluation of a defined word consists in the execution of each word composing its definition, sequentially (Figure 2).

Words are defined by compiling a sequence of symbols into the dictionary. The compiling operations are incorporated into the computation of some built-in words, such as the words : to enter compilation mode and ; to return to the interpretation state. For each word compiled into the dictionary, a numeric value called execution token (XT) is defined that points to the executable code of the word. The XT permits runtime computations on code to be executed. The word [’] (bracket-tick-bracket) is used in the definition of a new word to compile the XT of the following word in the chain as a literal. The word ’ (tick) has the same behavior at interpretation time so we use this word to build expressions referring to XTs. For instance, in Figure 2 the expressions ’ relay-open and ’ relay-close represent the XTs of the words relay-open and relay-close respectively, which are left on the stack by the execution of the word relay. This word-based programming paradigm gives the designer the possibility to develop code that is aligned to natural language syntax and semantics.

As an example, we consider an application including an actuation device, such as a lamp, connected to a relay on board the target hardware platform. A simple program to switch the lamp on could be:

lamp on

This would be actually a very natural choice for a Forth programmer. The goal expressed through this particularly clear semantics is reached, according to the computational paradigm, by executing the two syntactically independent words lamp and on, one after another. From the hardware perspective, switching the lamp on implies to drive the relay to close the circuit. Relays can be either normally-open or normally-closed and in some I/O expansion boards both kinds are provided. This two types are usually indicated in technical documentation with the abbreviations no and nc so that the two words can be naturally considered for inclusion in the dictionary. Hence, in such a case, the word lamp could be defined by executing the following compiling code:

: lamp no relay ;

where the words no and relay must have been previously defined. For the example depicted in Figure 2 the design choice is that the word no leaves on the stack the address of the normally-open relay. Then, the word relay pushes on the stack the XTs of two words switching the relay respectively on and off according to the relay address. The two hardware-dependent words, here defined as the words relay-open and relay-close, incorporate the low-level details, including the communication protocol over the specific hardware interface (e.g., GPIO, SPI or I2C). Finally, the word on could be defined with the code:

: on drop execute ;

The top-down design approach used for this example is shown in Figure 2.

**Figure 3:**
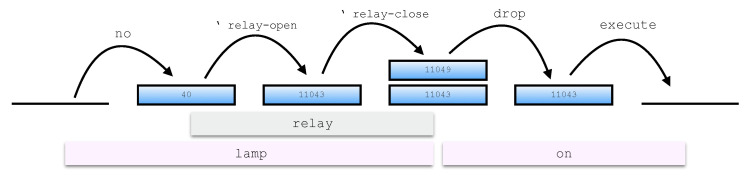
Stack effects of the execution of the sentence lamp on.

The word on drops the first item on the stack, which is the address of the word  relay-close, and then executes the topmost item, that is, the address of the code associated with the word relay-open, which finally switches the lamp on. The step-by-step stack effects of this implementation are provided in Figure 3. Similarly, the sentence led on could be used with an LED.

Due to the inherent complexity arising from not relying on a predefined syntax, the oracle problem for the symbolic paradigm provides a wide range of possible verification activities that go beyond mere input generation for unit testing and syntactic verification. An oracle, in this paradigm, is thus a sequence of words that: (i) is syntactically and semantically valid, and (ii) verifies both the state of the target hardware and of the physical environment are the expected ones. Generation of oracles and test cases is detailed in the next section.

## 4. System Overview

**Figure 4:**
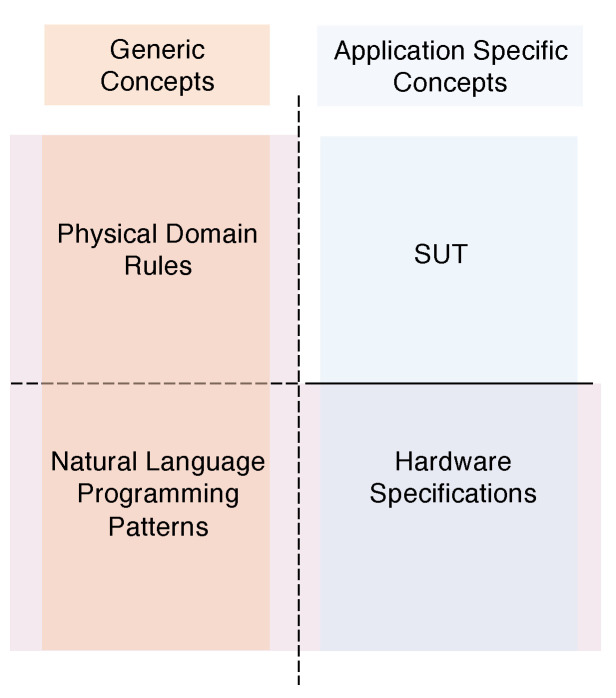
The three components of the knowledge base and the conceptual dependencies among them and the software under test (SUT).

The proposed system integrates knowledge about the environment, such as physical quantities and cause–effect relations, hardware specifications as well as syntax and semantics which model word sequences adhering to programming patterns inspired by natural language (Figure 4).

**Figure 5:**
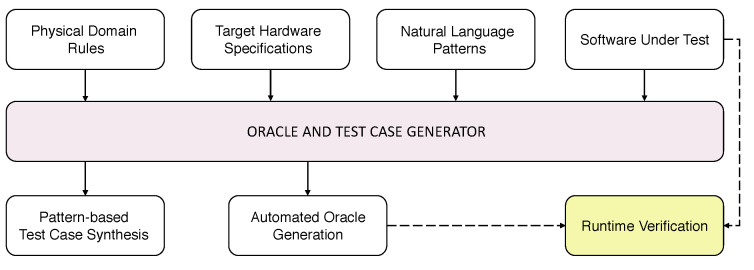
Architecture of the proposed automated test case and oracle generation system. The generated oracle is incorporated into the SUT for runtime verification on the target platform.

Rules defining physical domain concepts and natural language patterns are not tied to a specific application. Application code is instead strictly dependent on hardware specifications. However, the latter can be used to test all the SUTs running on the same hardware. Given the three distinct but closely tied components of the knowledge base, the system gets the SUT as input and generates the oracle, if it exists, to be incorporated into the SUT for its verification (see Figure 5). According to the computational paradigm described in Section 3, the input program is as a sequence of high-level words relating to the application domain. Similarly, the oracle is a sequence of words verifying hardware effects during the execution of the test.

Due to the nature of target applications, we focus on testing each actuating and sensing sentence as an independently verifiable component. For instance, in the following code:

$$\begin{array}{l} temperature\\ 50<if\\ \;\;\;\;\;\;green\;led\;on\\ \;\;\;\;\;else\\ \;\;\;\;\;\;green\;led\;off\\ \;\;\;\;\;then \end{array}$$

the sensing sentence temperature writes the value of the physical quantity to the top of the stack (TOS), then a comparison with a constant threshold value determines which of the actuation sentences is to be executed. The three sentences can be verified alone, and the correctness of the three components can also be assessed through a repeated execution test over a number of iterations (see Section 6).

Finally, given a programmer-defined word set, the system is able to generate test cases with word sequences that are syntactically and semantically valid because they adhere to natural language programming patterns specified in the knowledge base. Test cases and the respective oracles are then executed on the target platform implementing the symbolic computational paradigm. In the following, the components of the knowledge base as well as the oracle and test case generation are detailed.

### 4.1. Physical Domain Rules

**Figure 6:**
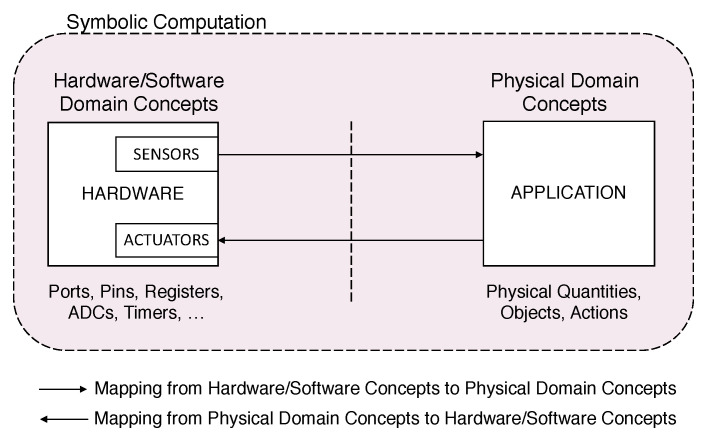
The Physical Domain is bidirectionally mapped to an internal hardware representation through sensing and actuation devices. Executable words of an application are defined in terms of lower level words concerning ports, registers, timers, and other hardware components.

The system includes the explicit specification of domain concepts and their relations. However, physical domain features are confined to those provided by sensing and actuation abilities of the hardware. In a broad sense, a system is specified in terms of its components, object classes, and interactions with the physical domain through sensing and actuation devices, as shown in Figure 6.

The same symbols used by rules to specify systems can also be defined as executable words. For instance, the word lamp not only defines a specific domain object but is also a symbol that is used in the high-level code running on the target hardware. The model including rules for an application consists of specifications about:

- **Objects and their features**. Classes of objects composing the system, e.g., lamps, LEDs, Heating Ventilation and Air Conditioning systems (HVAC), and so on, are defined as facts in the knowledge base. Object specifications also include class properties, possible states, and relationships with physical domain quantities.
- **Properties of the physical environment**. Unlike the approaches found in the literature [56], environmental features, which are physical quantities, are not described in terms of differential equations or continuous functions. Rather, ambient properties are confined to those measurable by the defined sensor devices.
- **Actuation**. High-level actions are described by means of their effects on the state of objects. As an example, a rule defining an action is:
  action([FinalState], [Object, _, FinalState]):- state(Object, FinalState).
  Each action is declared by binding a list of words describing it in natural language to a list containing an object name along with the initial and final states of the action. Considering the above declaration, the simplest way to change the object state is to indicate the final state to be reached, as with the command on. This action is independent of the initial state, thus the universal quantifier underscore (_) is used in place of a variable. The same applies to the definition including turn. The other definitions depend instead on both states so they are explicitly evaluated through named variables. Alternatively, the rules may include more complex natural-language sentences expressing possible actions having the same effects on the object state. Sentences that change (turn on, change state to on), or invert (invert state, toggle state). The definitions can be queried with either one or two instantiated arguments, to obtain the values satisfying the clause, if existing.
  Words composing sentences can be the same words used to build high-level sentences in the target programming language. Correct sentences are those satisfying actuation programming patterns.
- **Sensing**. Sensing allows to perceive the environment in terms of its physical quantities by reading the appropriate sensor devices. The simplest command to sense the environment consists in the name of the physical quantity to be measured. For instance, sensing may be expressed by simply using the physical quantity name, as in the following rule:
  perception([Physical],[Object, _]):- sense(Object,Physical).
  More structured sensing operations include commands to read a value or query a sensor expressed as list of words from natural language.
  Similarly to actuation, syntactically valid sentences, which include the same words used to specify sensing commands, are those satisfying programming patterns for sensing.
- **Qualitative dynamics of the system**. Actuation actions affect the physical domain state. These cause–effect relations are specified by rules that bind physical quantities to actuation abilities.

The knowledge base can be extended to include further objects, actions, perceptions, and events of the target application domain.

### 4.2. Hardware Specifications

Beside rules defining physical domain features, the oracle generation engine requires the hardware configuration as an input. From a structural point of view, the system is made of hardware components that are instances of classes of objects. For sensor devices, the hardware specification also includes the physical quantity they measure.

This description implicitly defines the physical quantities, which are considered environmental properties of the system, as a whole. Furthermore, it is possible to specify qualitatively hardware device properties as attributes, e.g., LED color, buzzer frequency, timer resolution, and so on.

The hardware specifications also incorporate the semantic mapping between actuation and sensing devices, and high-level expressions satisfying patterns that refer to objects. The same object can be in fact identified by its unique label or through one or more attributes that unambiguously distinguish it from other similar devices.

For instance, with respect to the specification provided in the knowledge base, the expressions led0 and green led are equivalent as they refer to the same device. The former expression identifies the object by name, while the latter provides a natural way to refer to the same device based on an attribute, that of emitting the green light. Similarly, sensor1 is equivalent to light sensor.

This equivalence results from rules that incorporate common-sense ways of referring to objects. In fact, when more instances of the same object class are present, an attribute is used as an object qualifier (e.g., green led vs. yellow led). Otherwise, the attribute is unnecessary as the instance can be uniquely referred to by simply using the name of its class. In case multiple objects have attributes with identical values, e.g., a system with two green LEDs, several choices are possible. The first is to refer to hardware components through their respective low-level identifier, which is in most cases straightly taken from the specifications (led0, led1). Otherwise, more attributes can be specified to distinguish one from another. For instance, in the case of the two green LEDs their function could be used as attribute value to signal a “ready” state with attribute(led0, ready) and a “loaded” state with attribute(led1, loaded), respectively. Finally, symbolic enumeration could be used to define a first green led and a second green led. The latter could be useful in a few circumstances, for instance to drive an LED bar-graph meter.

The system also provides specifications about actuation effects. Rules link an object to an actuation on it together with both the low level symbolic code to perform the action and code that verifies that the expected internal hardware configuration is reached. For instance, the low level verification code for actuation may include executable symbols related to hardware concepts such as porta or pin_input to set the data direction register.

For perception actions, the command to read the value enables the Analog to Digital Converter (ADC) (+adc), fetches its value to the TOS (adc@), then disables the ADC (-adc). In this case, the verification code fetches the value of the ADC on the TOS and checks that it equals the value that the previous execution of the command to be tested pushed below the TOS. A summary of low-level executable words used in the hardware effect specifications above is provided in Table A1.

The use of verification code in the oracle generation is detailed in Section 4.3, while the use of the hardware driving code to perform repeated test execution is discussed in Section 6.

### 4.3. Automated Oracle Generation

As described in Section 3, the implementation of the symbolic computation paradigm fosters the development of DSLs that directly drive the hardware without the need of multiple models and languages. As this approach naturally supports writing a program as a sequence of high-level words, we identified a set of programming patterns whose syntax and semantics are very close to natural language sentences.

Even if the SUT is verifiable, it is not sure that a behavior for the target system either exists or is as expected. In fact, the implementation of each word on the target machine is left to the programmer that defines the computation associated with the execution of each symbol.

For instance, there are several possible implementations for each of the words composing the sentence lamp on, although having the same hardware effect. Considering the implementation introduced in Section 3, running the word lamp implies the execution of the sentence no relay. The words relay and on may be defined as such:

$$\begin{array}{l} \;:\;relay\\ \;\;\;\;\;\;\;\;no?\;if\\ \;\;\;\;\;\;\;\;\;\;\;\;\left[’\right]\;no-open\;\left[’\right]\;no-close\\ \;\;\;\;\;\;\;\;\;\;\;else\\ \;\;\;\;\;\;\;\;\;\;\;\;\left[’\right]\;nc-open\;\left[’\right]\;nc-close\\ \;\;\;\;\;\;\;\;\;\;\;then\;;\\ :\;on\;drop\;execute\;; \end{array}$$

where the word no? checks if the normally open relay address is contained in the TOS. The word [’] compiles the XT of the following word as a literal. However, another implementation option is:

$$\begin{array}{l} :\;relay\\ \;\;\;\;\;\;\;parse-name\\ \;\;\;\;\;\;\;s"\;on"\;compare\;if\\ \;\;\;\;\;\;\;\;\;\;\;\;\;\;\;\;\;\;\;no?\;if\;no-open\;\;\;else\;\;\;nc-open\;then\\ \;\;\;\;\;\;\;\;\;\;\;\;\;\;\;\;\;\;else\\ \;\;\;\;\;\;\;\;\;\;\;\;\;\;\;\;\;\;\;no?\;if\;no-close\;else\;nc-close\;then\\ \;\;\;\;\;\;\;\;\;\;\;\;\;\;\;\;\;\;then\;; \end{array}$$

Differently from the previous implementation, the word relay parses the next symbol in the text input stream. If the symbol on is encountered –that is, the result of the comparison of the symbol and the constant string on left on the stack by s" on" equals to true– the relay is switched to close the circuit, otherwise the relay breaks it. Words no and nc are defined to respectively set or reset a boolean flag that is read and put on the TOS by the word no?. In this case, even if it is requested to complete a sentence the word on is not defined in the dictionary.

This example shows that, as different implementations are possible for the same high-level sentence, it is useful to incorporate mechanisms to verify that the program works correctly, i.e., the lamp reaches the on state, during its execution.

**Figure 7:**
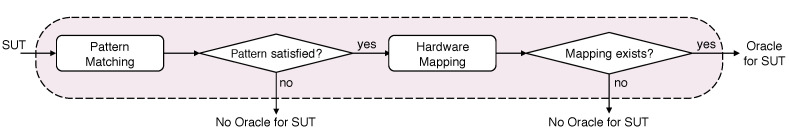
The oracle generation process.

Our system specifies the mapping between high-level object states and the corresponding internal hardware configuration, as described in Section 4.2. Hence, the system generates the oracle as the sequence of words to check that the actual hardware configuration meets the specified one. The oracle code is then embedded into the SUT while runtime verification occurs on the target platform. The oracle generation process is schematically shown in Figure 7.

Given a high-level sentence as the SUT, the first step includes syntactic and semantic verification to check that the sequence of words satisfies one of the specified patterns. If pattern matching fails, then the system will signal an inconsistency for that SUT. In this case there is no oracle. Otherwise, a high-level sensing, actuation or system sentence was recognized. A specific mapping between the high-level sentence and corresponding hardware is then looked for in the knowledge base. In the case of system patterns, the mapping includes code to verify the behavior of the network as a whole, by some algorithm for distribution of code or value collection. A failure at this point signals an inconsistency between the action, which is implemented by the sentence, and the expected high-level and hardware states. The oracle code is thus not generated. Otherwise, the oracle is generated and appended to the SUT code to be run on the target hardware.

The oracle generation does not involve any analysis of the word definitions designed by the programmer. Rather, the output code (SUT + oracle) is executed on the target machine and the result is placed on the parameter stack. A positive outcome indicates that the actual hardware configuration matches the specifications and suggests correct word implementations. For instance, given:

green led on

as SUT, the system would generate the following code:

green led on porta @ 2 and 0=

Code porta @ 2 and 0 = verifies the effect of the action by fetching the value of porta and performing a logic and operation with the bitmask value 2. If the value is equal to 0, then the pin is low and led0 is on.

At the end of the runtime execution the oracle would leave a truth value expressing the outcome of the test in the TOS. As the input sentence must meet the pattern, the high-level action in the input sentence, which was matched to it, must also match the respective hardware effect so that the corresponding oracle could be generated. Moreover, the object in the high-level action must be mapped to a hardware device. In essence, the oracle generation takes place by unification with the action and the object.

In the case of sentences adhering to system patterns, the oracle consists of a sequence of words to be run on single nodes assessing that the whole system task has been performed correctly. This requires nodes to be able to send symbolic code to peers, a feature unsupported by operating systems for resource-constrained devices. In our case, for the purpose we resort to the syntactic construct tell: :tell that we defined very easily in our experimental software platform due to the effectiveness of the computational model we adopted [53]. For instance, given:

network green led on

as SUT, the system would generate the following code:

network green led on bcst tell: porta @ 2 and 0=:tell

**Figure 8:**
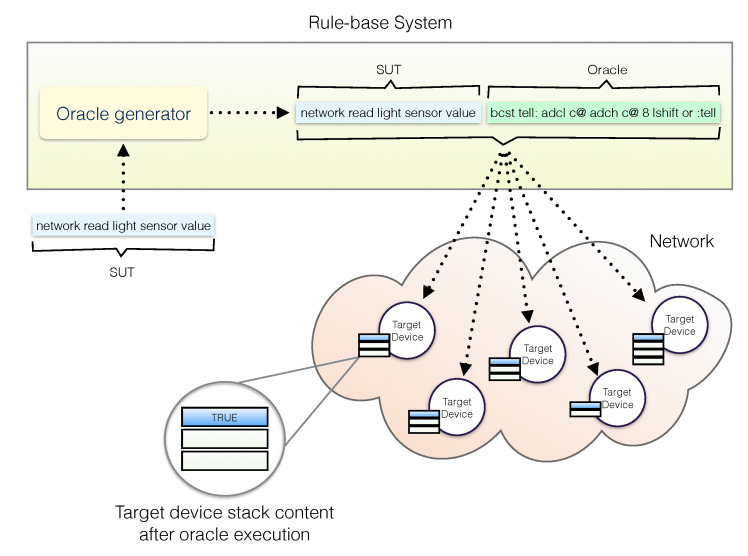
Network sensing task example. A SUT in form of a sentence is broadcast along with the generated oracle. A TRUE value on top of the target device stacks indicates that the programmer implemented the sentence correctly.

The oracle for a network task, for either actuation or sensing, consists of the sentence describing the task to be performed, i.e., green led on, followed by its oracle. In the example above, the oracle is enclosed between tell: and  :tell, all preceded by the broadcast address bcst. The oracle for the given SUT is executed by receiving nodes and the result of verification is placed on the stack of each node, as in the previous example. A sensing task example is provided in Figure 8.

In the case of a SUT performing an aggregation task at system level, as the following:

network light average

a sequence of words is generated and sent to remote nodes to compute the average light. At the end of the verification, the value from the averaging process implemented by the programmer and the value resulting from the execution of the verification code are left on the stack to be compared so to assess the correctness of the SUT.

### 4.4. Automated Test Case Generation

The proposed system exploits physical domain rules, and hardware and software specifications to automatically generate test cases.

**Figure 9:**
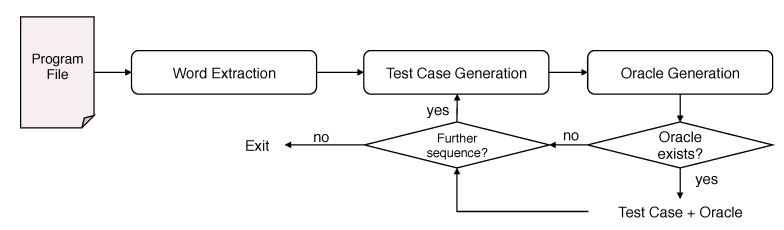
Test case generation. Given the word set defined by the programmer, the system generates all possible executable sentences along with the respective oracles.

The flowchart of the automated test case synthesis is shown in Figure 9.

Provided with the source code as a collection of word definitions, which have been designed by the programmer, the system uses the application domain word set as input. The first step thus just extracts only the names of each defined word from the source code. As a result of the unification mechanism, the system generates a collection of executable sentences that are obtained by exploiting all the possible word sequences. For each of these sequences, the system verifies that both syntactic and semantic specifications are met. Hence, valid test cases are sequences matching natural language-like patterns in the knowledge base. Each test case is provided as input to the oracle generator, as detailed in the previous section. If the oracle is not generated, then the sequence is removed from output test cases. Otherwise, the system incorporates the oracle into the test case. Test cases and the respective oracles are stored in separate source files and executed one at a time for runtime verification on the target hardware.

**Figure 10:**
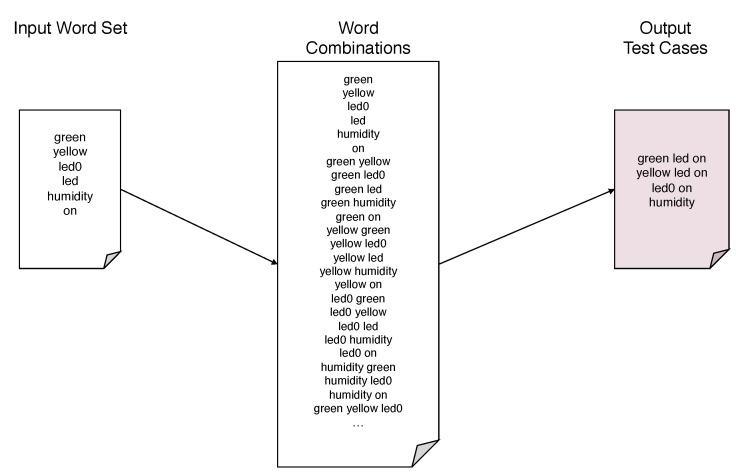
Given an input word set the system generates the test cases by combining the input words into executable sentences.

For instance, supposing that the programmer defined the domain-specific words green, yellow, led0, led, humidity and on, the system generates the test cases reported in Figure 10.

## 5. Ambiguous Semantics and High-Level Commands in Real Use Cases

As natural language is inherently ambiguous, building concatenative programming patterns inspired by natural language is not devoid of risks and pitfalls especially when defining commands for high-level tasks involving multiple devices. However, the spreading adoption of voice-based virtual assistants permitting the execution of commands in AmI and IoT applications signals that this is a path to be pursued. Indeed, the verification mechanisms of our approach mitigate some of the issues potentially arising in the design of programming patterns inspired by natural language. In the following, we discuss how semantic ambiguities are tackled in the proposed system, operatively. Then, we show the current system capabilities to verify high-level commands involving multiple devices.

As a first example, we consider potential ambiguities that may arise from the use of plural nouns. To define the plural form of nouns, the knowledge base holds facts such as plural(led,leds). As a consequence, the sentence

network green leds on

can only be interpreted as the command that turns all the green LEDs in the system on. On the contrary, the sentence

green leds on

is ambiguous as it is not easy to discern whether it addresses the system or a single device that has several green LEDs. Similarly, sentences including universal quantifiers could have multiple meanings:

all green leds on

Several disambiguation strategies can be considered. The first one is to specify two different patterns generating disjoint set of sentences. For instance,

green leds on

could indicate a command to be executed only locally, while

all do: green leds on

could specify a network command. A second strategy makes use of a pattern with two universal quantifiers. In this case, a valid sentence could be the following:

all all green leds on

The first quantifier would specify that the command refers to the whole network, while the second would indicate that all the green LEDs of a node are to be switched on. Another possible solution could be to let ambiguous sentences to include some context. However, we deliberately use context-free patterns as the implementation is left to the programmer.

Finally, the strategy that is adopted in this work is to solve the semantic ambiguity operatively. Thus, the sentence:

all green leds on

could satisfy either a system or a local pattern. Two oracles are associated to this sentence and are both generated by the rule system. The automatic generation of more than an oracle for a sentence warns the programmer about an ambiguity. Both oracles are then run on the target. The one associated to the system command interpretation is run first. If it succeeds, the green LEDs are turned off, then the local oracle is executed. The results of the oracles give the programmer useful information about the correspondence of the SUT execution with its design.

Sentences including plurals are executable only if more than one instance of an object is specified in the knowledge base. For instance, if two green LEDs are present, the oracle generation process for the sentence:

green leds on

produces the concatenation of the oracles for the sentences first green led on and second green led on. Anyway, generating possible oracles for ambiguous sentences allows the programmer to discriminate verification results occurred by chance or deliberately.

Sometimes, ambiguities are only apparent. For instance, consider the sentence:

green yellow and led on

In the natural-language interpretation the word and would be a postfix join operator. The sentence would then represent a command to turn both the yellow and the green LED on. However, in a programming language it would be unreasonable to give the word and meanings other than that of the boolean operator. Moreover, the low-level mechanism to turn the LEDs on, as showed previously, could consist in setting a couple of bits in an output I/O port register. The words green and yellow would simply push the respective bitmask on the stack. The sequence of words led on would then read the content of the I/O port register, apply the boolean OR to both values, and write the result to the I/O port register. Thus, the sentence would not be executable and it should rather replaced by:

green yellow or led on

The proposed system can generate and verify high-level commands involving many devices altogether, as found in real-life applications. In this context, the term “scene” is spreading in the IoT parlance to define a set of low-level commands leading to the desired configuration of a set of devices. For instance, executing the scene named “Good night” would turn all the lamps in a house off. In the following, we conform with this use of the term “scene” even if the related concept turns out to be much less central. In fact, instead of resorting to predefined scenes, our approach makes use of rules about the qualitative dynamics of the system. Given a set of devices, their initial state, and a high-level command, the system generates all the possible scenes to reach the final state. Scene code is produced by resorting to the prototypal hardware-driving code in the knowledge base. A scene is thus a low-level executable description of a state of the system. In general, multiple verifiable sentences share the same low-level scene code. The latter is thus used to group sentences producing the same hardware effect. As a consequence, all the high-level verifiable sentences associated to each scene can be generated along with the expected state and the oracle for each involved device.

As an example, we consider an environment including an LED, a lamp and a light sensor. A light increase may be produced alternatively by switching on either one or both light emitting devices. The system can be queried for all the scenes implementing the hardware effect of the increase light command as such:

$$\begin{array}{l} \mathrm{Devices}=[\mathrm{led}0,\mathrm{lamp}1,\mathrm{sensor}1],\;\mathrm{Goal}=[\mathrm{increase},\mathrm{light}],\;\mathrm{InitialState}=[\mathrm{off},\mathrm{off},\_],\\ \mathrm{scene}\_\mathrm{by}\_\mathrm{devices}(\mathrm{Devices},\mathrm{Goal},\mathrm{Sentences},\mathrm{Oracles},\mathrm{Scenes},\mathrm{InitialState},\mathrm{FinalState}). \end{array}$$

**Figure 11:**
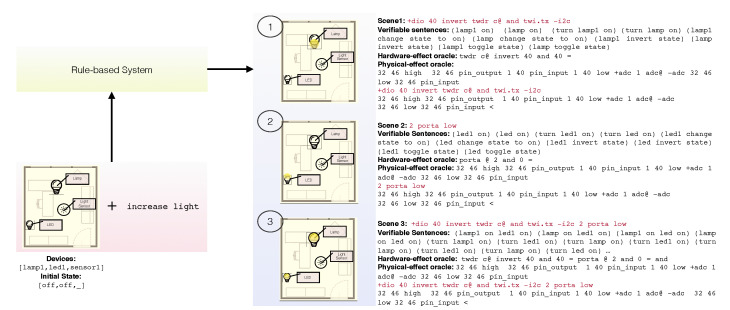
Given the set of hardware devices in the example, the initial state, and the high-level command increase light, the system generates three possible scenes. For each, all the verifiable sentences producing the same final state are also generated, along with the respective hardware-effect and physical-effect oracles.

The code for the generated scenes and the respective hardware-effect oracles is shown in Figure 11.

Besides hardware-effect verification, the causal relations modeled in the knowledge base permit to implement another verification mechanism for high-level commands based on their physical effects. In the example, a physical-effect oracle involves the feedback provided by the light sensor. In particular, the test oracle for [increase, light] encloses the scene code between the low-level code to acquire a light sample and the comparison code to assess the effective increase of light. The three physical-effect oracles generated for this high-level command are also reported in Figure 11.

## 6. Experimental Evaluation

This section describes the experimental results of the proposed system. In particular, tests evaluate both the formal system used for automatic oracle generation and the code generated by the formal system on the target machine. Concerning the formal specifications, the tests consist in (i) assessing the number of sentences which are executable and verifiable by the system with a given configuration of the knowledge base; (ii) evaluating the complexity of the specification code in terms of Lines of Code (LOC). On the other hand, as verification takes place onboard the target hardware, we also performed tests consisting in (i) generating repeated test execution code to verify the efficiency of a set of SUTs, which are usually required as commands in IoT applications, and (ii) evaluating the average execution time of a SUT on a target device.

Despite the fact that, for the sake of brevity, just a minimal set of concepts and hardware device specifications has been included in the test knowledge base, the number of valid sentences exceeds 1000. Sentences are grouped by pattern type and the number of valid sentences. The number of sentences which adhere to each of the considered patterns and that can be automatically verified by the system as an oracle exists are reported in Table 1. To evaluate the formal system upstream the oracle generation process, the knowledge base was partitioned into components, as reported in Figure 4 and LOC was measured for each of them. Results concerning the specification system code complexity are provided in Table 2.

The second evaluation is about how the rule-based system can also be used to generate code that performs repeated test execution on the target hardware. Beside verifying correctness of code implementation, repeated tests can be used to assess the efficiency of SUTs with respect to reference low-level code, which is the hardware-driving code, stored in the knowledge base.

To this purpose a natural language-like sentence is given as a input to the system and an oracle is generated, as described in Section 4.3, and appended to the sentence. The resulting code (SUT + oracle) is included in a loop that executes it #Repetitions (number of repetitions) times along with code updating statistics concerning correctness with respect to the effects on the hardware and execution time. For the correctness assessment the count of failed verifications is recorded. This loop is contained in another one that makes it execute for values of #Repetitions ranging from 1 to #MaxRepetitions (maximum number of repetitions) with a Step increment. Both #MaxRepetitions and Step are chosen by the tester. For each iteration of the external loop the statistics are collected and associated with the value of #Repetitions. The collected data are useful to verify the correctness of the high-level code, highlighting issues triggered by increasing repetitions of the sentence like stack overflows, memory leakages, unresponsive subsystems, or conflicts with interrupt service routines. Estimates of the asymptotic trend of the execution time can also be easily plotted.

An execution time baseline estimator can also be generated that exploits the hardware specifications replacing the high-level sentence with the related sequence of low-level symbols defined in the knowledge base (see HWDrivingCode in Section 4.2). Provided that this code has been crafted as efficient as possible, driving the hardware through low-level words, the estimator provides the lower bound for the execution time, as application code is destined to trade expressiveness and abstraction for execution time. Considering the simplest sentence for this purpose (light) according to the defined patterns (see Section 4.1), the rule base system is queried to generate the repeated test code as follows:

SUT=[light], Step=100, repeated_execution_test_code(SUT,Step,TestCode).

**Figure 12:**
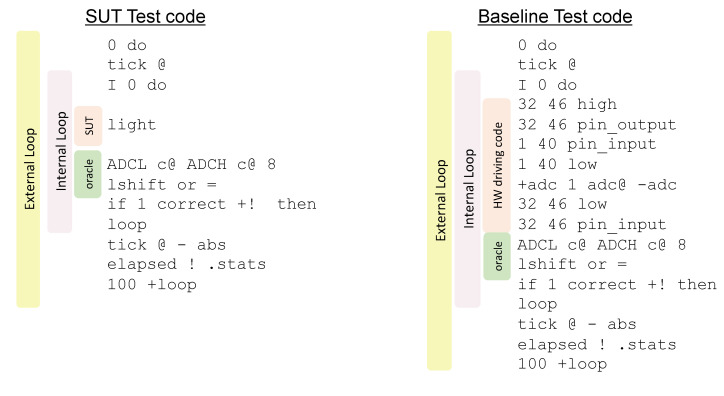
Repeated execution test codes for a high-level sentence to acquire a light sample. The high-level hardware-driving code and its oracle is put in the internal loop and statistics are updated. The external loop increments the upper limit of the inner loop by 100. The word tick acquires the current value of the timer. The timer value is fetched at the start and at the end of the internal loop to obtain the execution time by difference.

**Figure 13:**
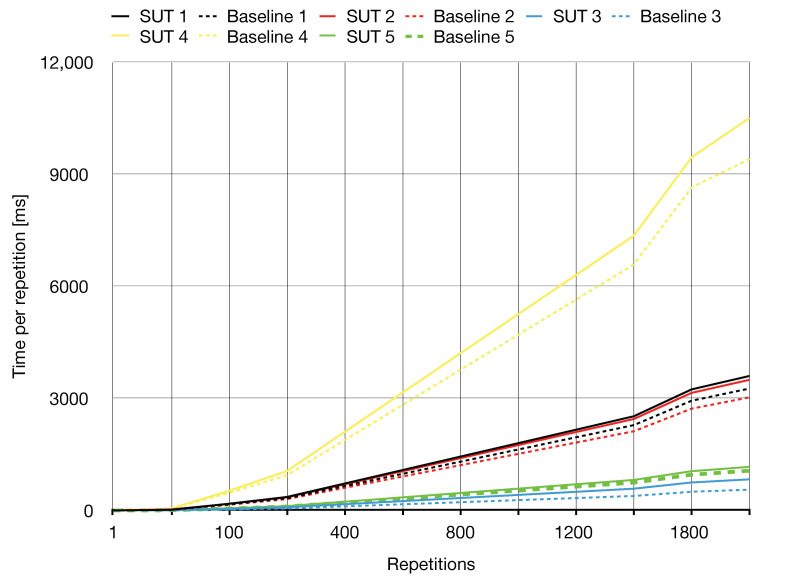
Results of repeated test execution for the benchmark SUTs.

The generated test code is provided in Figure 12. The code requires the upper bound (#MaxRepetitions) to be on the stack before execution. The step increment (Step) is 100. The sensing operation leaves the sensory reading in the TOS. The target hardware for our tests was an IRIS WSN node running the AmForth (v. 5.4) Forth environment [57]. As the light sensor on the target device is connected to the ADC, the oracle consisted in the word sequence to read the ADC register and to compare its content with the TOS. In the following, this example SUT is addressed as benchmark SUT 1. Results of the repeated test execution with the upper limit of 2000 repetitions are reported in Figure 13 (benchmark SUT 1) along with the results of all the other benchmarks. The system behaved correctly for any repetition number. As expected, the baseline time was slightly lower than the execution time on the target platform. This difference, amounting to a few CPU cycles, is easily ascribed to the indirection code included in the definition of light, which is missing in the inlined definition of the Baseline test code. As seen in the graph plot, this difference would be actually measurable with rather low precision on the target hardware, due to the relatively large granularity of the timescale of the timer circuit with respect to the execution time of the indirection.

The same evaluation was carried out for the representative set of SUTs collected in Table 3. Results are provided in Table 4. The zero values for test with #Repetitions=1 are again ascribable to the precision of the on-board timer circuit.

Finally, precise estimates of the execution time of the SUT were obtained by repeating execution over the maximum number of iterations (2000) used for the repeated execution tests. This evaluation provides an assessment about the average execution time, which is typically desirable on IoT applications with time constraints. Indeed, the programmer can benefit from an estimate about the execution cost of each command on the real target hardware before deployment. Estimates for a representative set of benchmark SUTs were measured and reported in Table 5. Finally, results also show that by adopting the repeated execution methodology for tests on-board estimation of execution efficiency of high-level code can be performed on resource-constrained devices with as low as 128 KB of storage memory, 8 KB of RAM, and a 10 MHz CPU.

## 7. Conclusions

Design and verification of applications interacting with physical environments is challenging as it requires the programmer to combine models and languages to treat different domain aspects while dealing with resource constraints. Novel programming paradigms and high-level abstractions are needed to address these issues and support the interoperability required by AmI and IoT scenarios.

In this paper we proposed a knowledge-based methodology for the development of applications as sequences of words matching natural language patterns. The approach exploits a simple but effective, even on resource-constrained devices, stack-based symbolic computational model and a knowledge base integrating specifications about physical domain, hardware specifications, and programming patterns. The knowledge base explicitly specifies the mapping between recurring operations, such as sensing and actuation, and the corresponding hardware. We also showed how the knowledge base can be easily extended to accomodate more complex patterns that describe commands for a system or a network as a whole. An oracle and test generation system for runtime verification of applications on the target hardware was also presented. Although the oracle generation is offline, verification is performed on the target platform, differently from other approaches in the literature. Test generation produces code to verify that program components behave as expected in repeated executions.

The proposed methodology supports the definition of DSLs that are verifiable on independent hardware devices to be integrated into larger systems. These DSLs have clearly defined boundaries with respect to the domain included in the knowledge base but do not force software designers to choose a specific syntax. Moreover, due to the simplicity of the adopted computational model, the defined DSLs can be used on both resource-rich and resource-constrained nodes. Furthermore, as symbols themselves represent domain concepts, interoperability can be pursued without recurring to complex ontologies. In fact, symbols can be directly taken from natural language and combined according to common sense patterns. Software designers adopting the Forth methodology are used to building such patterns, usually through several iterations of refactoring. Future work will investigate recurrent operations and interactions among nodes, addressing communication patterns for distributed programming. Other possible future extensions include time intervals as well as non imperative patterns. With respect to system patterns, other extensions will be evaluated, for instance to partition nodes in groups satisfying given conditions.

## Figures and Tables

**Table 1:** Executable sentences for the test knowledge base.

		Number	Example
**Local**	Sensing	45	light, humidity, infrared, sensor1 sample, light sensor sample, sensor2 sample, get light sensor sample, get sensor1 value, query light sensor, read humidity sensor, ...
	Actuation	80	led0 on, led1 on, first green led on, second, green, led on, led0 off, led1 off, turn first green led on, ...
	Aggregation	36	humidity average, humidity maximum, humidity minimum, humidity samples average, humidity, samples maximum, humidity samples minimum, light average, ...
	Time	23	seconds, milliseconds, current time in milliseconds, current time in seconds, elapsed, get milliseconds, get seconds, ...
**System**	Sensing	180	network light, network light sensor sample, all do get humidity sensor sample, all first green led on, network light average, ...
	Actuation	320	network led0 on, network first green led on, all 4KHz buzzer change state to off, network lamp on, all lamps on, all nodes do lamp off, all do turn 4kHz buzzer off, ...
	Aggregation	144	network humidity average, network humidity maximum, network humidity minimum, all node do humidity samples average, network humidity, network samples maximum, network humidity samples minimum, all node do light average, ...
	Time	92	all seconds, all milliseconds, network seconds, all do current time in milliseconds, network current time in seconds, network get milliseconds, all get seconds, ...
**Plural**	Local	31	green leds on, leds on, green leds off, buzzers change state to off, buzzers change state to, on, get sensors value, ...
	System	155	network green leds on, network leds on, all buzzers change state to off, all get sensors sample, all read sensors, all buzzers off, all do sensors sample, ...
	**Total**	1106	

**Table 2:** Knowledge base code complexity in terms of Lines of Code (LOC).

Component	LOC
Physical Domain Rules	85
Hardware Specifications	55
Natural Language Programming Patterns	57
Automated Oracle and Test Case Generation	30
High-Level Commands in Real Use Cases	11
Total	238

**Table 3:** Benchmark SUT number and the respective code.

Benchmark Number	SUT Code
1	light
2	lamp on
3	alarm on
4	light 400 < if lamp on else lamp off then
5	time @ elapsed 5 < if all leds on else all leds off then

**Table 4:** Time per repetition [ms] for a set of benchmark SUTs and the respective baseline test code.

Benchmark Number	Description		#Repetitions								
		Time per #Repetitions [ms]	1	10	100	400	800	1000	1400	1800	2000
1.	Acquire a light sample	SUT Test Code	1	18	180	720	1441	1800	2521	3241	3600
		Baseline Test Code	1	17	163	652	1305	1631	2284	2937	3263
2.	Switch a lamp on	SUT Test Code	2	28	175	700	1399	1749	2448	3148	3497
		Baseline Test Code	2	15	152	605	1211	1514	2119	2725	3028
3.	Fire an alarm	SUT Test Code	0	5	42	167	333	416	583	749	833
		Baseline Test Code	0	3	28	111	222	278	389	500	555
4.	Turn the lamp on if the belowis under a certain value	SUT Test Code	5	52	525	2101	4202	5252	7356	9452	10,503
		Baseline Test Code	4	47	470	1880	3763	4702	6582	8645	9404
5.	Turn all the LEDs on if elapsedtime is over a threshold	SUT Test Code	1	6	59	233	467	584	817	1051	1167
		Baseline Test Code	0	5	53	213	425	531	744	957	1063

**Table 5:** Average Execution Time over 2000 repetitions for a set of benchmark SUTs along with their respective baseline test code.

Benchmark Number	Average Execution Time [ms]		Efficiency Ratio (SUT/baseline)	Number of Words	
	**SUT**	**Baseline**		**SUT**	
1.	1.8	1.63	1.1	16	
2.	1.75	1.51	1.16	17	
3.	0.42	0.28	1.5	14	
4.	5.25	4.70	1.12	55	
5.	0.58	0.53	1.10	45	

## Data Availability

Not applicable.

## Appendix A

**Table A1:** Summary table of a subset of low-level words used by specification rules in Section 4.2. Standard Forth words are described in the upper part of the table, hardware-dependent words in the lower.

Word	Description
@	Fetch a memory word from the address left on the TOS
c@	Fetch an 8-bit value from the address left on the TOS
!	Store the second item on the stack to the address in the TOS
+!	Increment the content of the address on the TOS by the second item on the TOS
<>	Leave true on the stack if the two topmost items on stack are different
=	Leave true on the stack if the two topmost items on stack are equal
abs	Compute the absolute value of the number in the TOS
and	Logical and between the two topmost item on the stack. Result is written to TOS
do	Start a defined iteration. The upper and lower bound must be on the stack
I	Push the current value of the iteration counter on the stack
if	If the value in TOS is true execute the following words until the word then is reached,
	otherwise skip past them
+loop	Increment the current value of iteration by the number in the TOS
lshift	Using the two topmost values (n, k) on the stack perform a logical shift left of k
	bits-places on n leaving the result in the TOS
or	Logical or between the two topmost item on the stack. Result is pushed on TOS
s¨	Parse the next sequence of chars until ¨ is encountered. The string address and
	its length are left on the stack
swap	Swap the two top elements of the stack
then	Conclude the selection construct and continue execution
ADCL	Leave the address of the IRIS mote ADCL data register (low byte) in the TOS
ADCH	Leave the address of the IRIS mote ADCH data register (high byte) in the TOS
+adc	Enable the ADC
adc@	Fetch the content of ADC writing it to the TOS
-adc	Disable the ADC
low	Turn a port pin off
high	Turn a port pin on
porta	Leave the address of target hardware port PORTA in the TOS
pin_input	Set a port Data Direction Register (DDR) pin as input
pin_output	Set a port Data Direction Register (DDR) pin as output
+dio	Initialize the I2C subsystem and send the address of the 8-channel digital I/O
	expander on the MDA300 data acquisition board
twdr	Leave the Two Wire (I2C) Data Register (TWDR) address on the stack
twi.tx	Send one byte through I2C
-i2c	Send stop condition through I2C
